# Commentary: Differential Signaling Mediated by ApoE2, ApoE3, and ApoE4 in Human Neurons Parallels Alzheimer's Disease Risk

**DOI:** 10.3389/fnagi.2020.00127

**Published:** 2020-05-08

**Authors:** Patrycja Dzianok, Ewa Kublik

**Affiliations:** Laboratory of Emotions Neurobiology, Nencki Institute of Experimental Biology PAS, Warsaw, Poland

**Keywords:** Alzheimer's disease, APOE, neurodegeneration, synapse, risk-genes

Alzheimer's disease (AD) is a neurodegenerative condition that inevitably impairs cognitive functions and influences a patient's behavior, mood, and self-reliance. Due to demographic changes, AD and other age-associated diseases have become increasingly common and burdensome for families, as well as entire societies. It is extremely important that we learn more about specific mechanisms that can be linked to the development of the disease. The main symptoms of AD, observed in the central nervous system, are brain atrophy and loss of neurons and synapses. They are believed to result from excessive aggregation of tau protein and amyloid plaques (composed of ß-amyloid). However, neither the initial cause nor the detailed chain of events that lead to this type of neurodegeneration are known. No deterministic genes were identified for late-onset Alzheimer's disease (LOAD), but several risk genes seem to be involved in its pathogenesis. The gene coding apolipoprotein E (*APOE*) is the best-known and has the strongest association with AD development. AD probability decreases in carriers of the e2 variant of the *APOE* gene (*APOE-*e2), whereas *APOE*-e4 is believed to be a strong risk factor (Strittmatter et al., 1993) and is associated with overall cognitive impairment and synapse loss (see review by Selkoe, 2002).

Few hypotheses have been proposed in the literature explaining possible mechanisms by which *APOE* could affect the brain and promote AD. ApoE in the brain is mostly expressed by astrocytes and microglia and is thought to be involved in the metabolism and clearance of lipoproteins (see Fernandez et al., 2019 for review). Astrocytes play a vital role in the internalization and degradation of extracellular beta-amyloid (Aβ), the component which forms plaques that are believed to be involved in AD neurodegeneration (Serrano-Pozo et al., 2011; Ries and Sastre, 2016, see also review by Haass and Selkoe, 2017; Fernandez et al., 2019). The *APOE*-e4 variant was shown to be least effective in degradation of Aβ (Castellano et al., 2012).

Another hypothesis points to the fact that ApoE variants have different binding properties (Calandra et al., 2011) to the receptors that regulate intracellular signaling (Ohkubo et al., 2001; Qiu et al., 2004). This hypothesis was first addressed by Huang et al. (2017) and again in a replication and control study published last year in the Journal of Neuroscience (Huang et al., 2019). Their research was conducted on stem cell-derived human neurons cultured without glial cells. Results of the experiments (Huang et al., 2019) showed that even in the absence of glial cells ApoE strongly and diversely influenced signal transduction cascades in neurons, which led to intensification of amyloid precursor protein (APP) synthesis and, at the same time, to the formation of new synapses. The study revealed the synaptic paradox of the *APOE*-related risk of AD: surprisingly, it was *APOE-*e4, the gene variant that is linked to the highest risk of AD, that was most efficient in stimulating MAP signaling and in enhancing synaptogenesis.

The question arises: how is it possible to link these cell-level studies with the same ranking (*APOE-*e4 > *APOE-*e3 > *APOE-*e2) of negative impact on human brain function in AD. Results indicating enhanced APP synthesis are in agreement with studies showing higher levels of Aβ in brains of *APOE*-e4 carriers, examined post-mortem (Shinohara et al., 2013), as well as *in vivo* studies using positron emission tomography (see review of Jack and Kepe, 2013). However, a reported *APOE*-e4-related increase in synapse formation contradicted numerous findings indicating the highest loss of synapses and severity of cognitive decline in *APOE-*e4 carriers (Terry et al., 1991; Selkoe, 2002; Scheff et al., 2006; Purro et al., 2012; Chen et al., 2018). It appears that the link between *APOE* isoforms and neuronal and synaptic dysfunction observed in AD comprises multiple, seemingly contradictory, mechanisms. Huang et al. (2019), and previously Lin et al. (2018), showed an *APOE*-e4 related increase in the number of synapses in isolated neurons. On the other hand *in vivo* research has demonstrated strong evidence of synapse loss related to memory and cognitive impairment, which characterize dementia and neurodegeneration. The direct effect of *APOE-*e4 on neurons can be modulated by the interplay of many factors, including the activity of glial cells (mainly astrocytes and microglia) and other risk-genes. Moreover, it was shown that neurons need astrocytes and microglia to eliminate redundant synapses (Lee and Chung, 2019). Maintaining proper synapse number is a crucial process in learning and memory and thus any changes may disrupt the cells' homeostasis and lead to neurodegenerative diseases like AD. *APOE-*e4 was shown to inhibit synaptic pruning, realized by astrocyte phagocytosis, whereas *APOE*-e2 promoted it Chung et al. (2016). *APOE* has a strong impact on lipoprotein (and cholesterol) homeostasis and synaptic stability maintenance, with *APOE-*e4 having the most negative impact on the brain. *APOE*-e4 limits the astrocytes' ability to recycle and clear extracellular cholesterol (Fernandez et al., 2019) and leads to its accumulation and an increase of Aβ (Strittmatter et al., 1993) related to synaptic dysfunction (Purro et al., 2012). Human astrocytes with *APOE-*e4 showed accumulation of cholesterol and could not efficiently fulfill their role related to clearance of Aβ (Lin et al., 2018).

Perhaps it is the initial higher number of synapses and APP in neurons with *APOE*-e4 that leads to an increase in toxic Aβ forms and impairs astrocytes' function, which can initiate the whole cascade of changes related to later loss of synapses and cognitive functions. It may indicate that, in the brains of *APOE*-e4 carriers, AD risks begin to accumulate from early developmental stages when too many synapses are formed and not enough of them are pruned (Chung et al., 2016; Lin et al., 2018; Huang et al., 2019). Although, the *APOE-*e4 risk related to loss of cognitive functions is predominant for persons older than 50 years of age, people homozygous for *APOE-*e4 may experience the risk much earlier, just after 40 years of age (Liu et al., 2010). Other studies point out that *APOE-*e4 could affect the brain even earlier, changing its structure, function and neurochemistry (see DiBattista et al., 2016 for review). *APOE*-e4 young carriers perform equally well or even much better in a variety of cognitive tasks compared to non-carriers (Mondadori et al., 2007; Jochemsen et al., 2012; DiBattista et al., 2016). The effect is tried to be explained by antagonistic pleiotropy hypothesis: some genes may enhance fitness early in life but act adversely in elderly. They are still favored by natural selection since the survival of the species depends on young individuals (Tuminello and Han, 2011; DiBattista et al., 2016). Understanding the specific influence of *APOE*-e4 on neuronal signaling pathways throughout the lifespan may help us to identify early biomarkers and target therapy against AD in the future.

## Author Contributions

PD wrote the first draft. EK critically edited and improved the manuscript. PD and EK read and approved the final version of the manuscript.

## Conflict of Interest

The authors declare that the research was conducted in the absence of any commercial or financial relationships that could be construed as a potential conflict of interest.
